# The path of voices in our brain

**DOI:** 10.1371/journal.pbio.3001742

**Published:** 2022-07-29

**Authors:** Benjamin Morillon, Luc H. Arnal, Pascal Belin

**Affiliations:** 1 Aix Marseille University, Inserm, Institut de Neurosciences des Systèmes (INS), Marseille, France; 2 Institut de l’Audition, Inserm unit 1120, Institut Pasteur, Paris, France; 3 Aix Marseille University, CNRS, La Timone Neuroscience Institute (INT), Marseille, France

## Abstract

Categorising voices is crucial for auditory-based social interactions. This Primer explores a PLOS Biiology study that capitalises on human intracranial recordings to describe the spatiotemporal pattern of neural activity leading to voice-selective responses in associative auditory cortex.

## Teaser

Categorising voices is crucial for auditory-based social interactions. A recent study by Rupp and colleagues in *PLOS Biology* capitalises on human intracranial recordings to describe the spatiotemporal pattern of neural activity leading to voice-selective responses in associative auditory cortex.

The voice is the main carrier of human communicative signals. Thanks to the unique acoustic attributes of vocal signals, we can not only very quickly distinguish conspecifics from any other natural sounds, but also extract complex information regarding the identity, the emotional state, the communicative intent, and the meaning of the emitter’s utterances. Just hearing the syllable “Ah!” is enough to guess the size, gender, emotional state, and identity of a speaker. As such, categorising voices constitutes a primary and crucial processing step for auditory-based social interactions.

**Fig 1:**
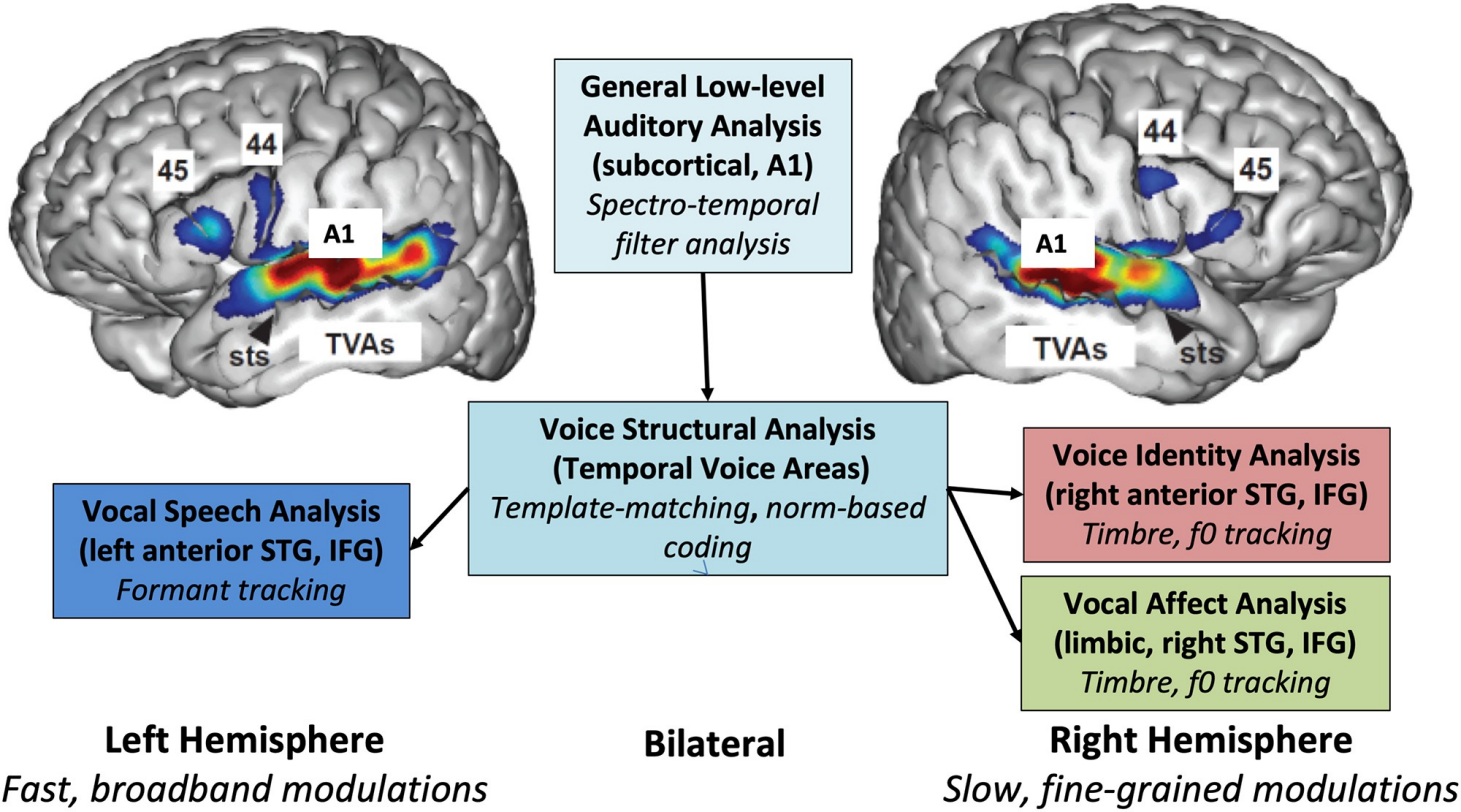
The functional processing hierarchy of auditory communicative signals. TVAs are highlighted. They are the critical intermediate processing stage between general auditory analyses and hemispherically lateralized processes dedicated to socially relevant auditory signals. IFG, inferior frontal gyrus; STG, superior temporal gyrus; STS, superior temporal sulcus; TVA, temporal voice area.

A new publication by Rupp and colleagues in *PLOS Biology* [1] capitalises on human intracerebral recordings of individuals with epilepsy implanted for clinical purposes to further examine how voices are categorised by the human brain. Voices constitute a distinctive auditory category that selectively activates specific “voice patches” in bilateral associative auditory cortex: the “temporal voice areas” (TVAs; see Fig 1; [2]). Such category-selective auditory responses have recently been also described for music, and even songs [3]. Here, the authors show that even in the complete absence of linguistic content, voices are categorically processed in anterior areas of the superior temporal gyrus/sulcus (STG/STS), in line with the fundamental role of voices in communication. This selectivity for conspecific voices is also found in nonhuman primates [4]. This phenomenon points towards evolutionary conserved principles of efficient coding of socially relevant stimuli—as assumed for faces—by expert brain regions dedicated to fine-grained discrimination of perceptually similar stimuli [5].

Intracranial EEG signal provides temporally precise information about the functionally selective engagement of neuronal populations at the millisecond scale, which is necessary for accurately depicting the neurophysiological underpinning of a specific cognitive process. While functional MRI has been used in previous studies to promote a spatial code of voice encoding, these new results extend this model by integrating the temporal dimension. Voice-selective neural responses are sustained throughout the stimulus duration and even last after stimuli offsets (approximately 500 ms). Future work may further decipher the spatiotemporal structure underlying neural selectivity (i.e., the internal model of voices; see below) in terms of representational dynamics [6].

The authors also show that while primary auditory regions encode acoustic features of varying complexity (loudness, spectral flux, etc.) and can be modelled with purely acoustic parameters (see also [4]), a voice/nonvoice categorical component is needed to best model responses in associative auditory regions. Previous work suggests that a template matching, “norm-based coding” phenomenon is probably at play. In this view, neural responses reflect not the stimulus itself but rather how well it matches an internal template (a norm), possibly averaging our personal experience of voices accumulated within our social context [7]. However, the reason why humans can so easily detect and recognise voices from other sounds is because they use distinctive acoustic features. Recent works have shown that communicative signals (e.g., alarm, emotional, linguistic) exploit distinct acoustic niches to target specific neural networks and trigger reactions adapted to the intent of the emitter [8,9]. Using neurally relevant spectrotemporal representations, these works show that different subspaces encode distinct information types: slow temporal modulations for meaning (speech), fast temporal modulations for alarms (screams), spectral modulations for melodies, etc. Although the authors account for a variety of acoustic attributes in their modelling of the data, which features—and which neural mechanisms—are necessary and sufficient to route communicative sounds towards voice-selective modules in the temporal cortex remain open questions.

Interestingly, while voice patches are observed bilaterally in the auditory associative areas [1,2], processing of familiar voice–identity recognition is largely a right-lateralized process [10]. This distinction is also observed in other cognitive domains, such as speech and melodies. While selective responses to voice and music categories occur bilaterally in associative auditory regions [3], processing of sentences and melodies, respectively, occur in the left and right associative auditory cortex [9]. This lateralisation arguably reflects the complementary specialisation of 2 neural systems functioning in parallel in each hemisphere to maximise the efficiency of encoding of their respective acoustical features. In the context of social auditory communication, the stages of voice analysis are sequentially anchored in the hierarchy of auditory processing. Starting bilaterally with the rapid identification of the relevant cognitive domain (here auditory communication), the routing of vocal information obeys a functional division of labour entailing the lateralized specialisation of anterior temporal regions for the parallel processing of complex social affordances (i.e., meaning, affect, and identity).

Here, the authors investigate how the brain encodes voices (compared to nonvoice stimuli), but not how each voice identifies individuals, although this aspect is a hallmark of voice recognition, together with linguistic and emotional information (see Fig 1). Whether the specialised voice-processing function identified in the literature extends to distinguishing conspecifics’ identity was not tested. Future work could use dedicated classification analyses to help decipher whether individual identification occurs at this level or at downstream levels.
